# Immune Modulatory Profile of the Pateamines PatA and Des-Methyl Des-Amino PatA

**DOI:** 10.3390/ijms252111430

**Published:** 2024-10-24

**Authors:** Susanne Schiffmann, Marina Henke, Sophie Brünner, Alexandre Bennett, Yassin Yagubi, Francesca Magari, Michael J. Parnham, Arnold Grünweller

**Affiliations:** 1 Fraunhofer Institute for Translational Medicine and Pharmacology ITMP, Theodor-Stern-Kai 7, 60596 Frankfurt am Main, Germanymike.j.parnham@gmail.com (M.J.P.); 2 Faculty of Medicine, Institute of Clinical Pharmacology, Goethe University Frankfurt, Theodor-Stern-Kai 7, 60590 Frankfurt am Main, Germany; 3 Institute of Pharmaceutical Chemistry, Philipps-University Marburg, Marbacher Weg 6, 35032 Marburg, Germany; magari@staff.uni-marburg.de (F.M.);; 4 EpiEndo Pharmaceuticals ehf, Bjargargata 1, 102 Reykjavik, Iceland

**Keywords:** immune modulation, pateamine A, des-methyl des-amino PatA, eIF4A, rocaglates, translation initiation, energy metabolism, cytokines

## Abstract

Pateamines act as inhibitors of the RNA helicase eIF4A and exhibit antiviral and anticancer properties. Recently, we observed that inhibition of eIF4A by rocaglates affects the immune response. To investigate whether the observed immunomodulatory effects are specific to rocaglates or the inhibition of eIF4A, a comprehensive study was conducted on the influence of pateamines that exhibit the same inhibitory mode of action as rocaglates on various immune cells. The effects of pateamine A (PatA) and des-methyl des-amino pateamine A (DMDA) on the expression of surface markers, release of cytokines, cell proliferation, inflammatory mediators and metabolic activity in primary human monocyte-derived macrophages (MdM), T cells and B cells were assessed. Additionally, safety and bioavailability profiles were determined. DMDA revealed almost no immunomodulatory effects within the tested concentration range of 0.5–5 nM. PatA reduced B cell activation, as shown by reduced immune globulin release and decreased chemokine release from macrophages, while T cell function remained unaffected. Both DMDA and PatA showed low permeability in Caco-2 and Calu-3 cell barrier assays and no mutagenic potential. However, 10 nM PatA exhibited genotoxic potential, as shown by the micronucleus assay. In conclusion, DMDA had a good safety profile but exhibited low permeability, whereas PatA had a poor safety profile and also low permeability. The observed immunomodulatory effects of elF4A inhibitors on B cells appear to be target-specific.

## 1. Introduction

The RNA helicase elF4A is a component of the heterotrimeric translation initiation complex elF4F, responsible for the unwinding of stable RNA structures to facilitate ribosome binding, thereby initiating translation. elF4A plays a crucial role in cellular processes such as growth and proliferation. Inhibitors of the elF4A helicase have been investigated for their potential anticancer effects [1]. Moreover, the elF4A helicase is important for replication of several virus families, including flavi- and coronaviruses [2,3].

Specific and potent elF4A helicase inhibitors include rocaglates such as silvestrol, CR-31-B (-) and zotatifin, as well as pateamines. Pateamines exhibit a less restrictive binding mode to the eIF4A-RNA complex, in terms of interaction with specific amino acids, compared with rocaglates [4]. Pateamines clamp RNA onto the surface of eIF4A, showcasing a unique mechanism [5]. As with rocaglates, they might be expected to specifically inhibit the eIF4A-RNA complex, with the only known secondary target being the RNA helicase DDX3 [6]. Due to their distinct mode of action (RNA clamping), pateamines and rocaglates exhibit functional mimicry that has evolved independently in two different organisms: plants of the genus *Aglaia spec* and the marine sponge *Mycale hentscheli* [7].

While silvestrol and the pateamine PatA are natural compounds, synthetic rocaglates (e.g., CR-31-B (-), zotatifin) or synthetic pateamines like des-methyl des-amino-PatA (DMDA) were developed. All of these elF4A inhibitors have shown antiviral activity against several virus strains in the nanomolar range [3,4,8]. Consequently, eIF4A inhibitors are potentially broad-spectrum antivirals.

However, besides an acceptable safety profile, silvestrol has shown low bioavailability and an immune modulatory potential [9]. Specifically, silvestrol prevented B cell and dendritic cell activation, indicating an immune-suppressive potential for silvestrol [10,11]. Zotatifin is already in clinical trials [Intravenous Zotatifin in Adults with Mild or Moderate COVID-19 (PROPEL), NCT04632381], indicating acceptable bioavailability and safety. Another advanced synthetic rocaglate is CR-31-B (-), which also prevented B cell activation and T cell proliferation, suggesting an immune-suppressive potential. This suggests that rocaglates, while inhibiting viral replication, may also suppress bystander tissue injury by the host immune system or the host’s immune response against the virus. The first could be beneficial, while the latter could be detrimental. The previously studied rocaglates (e.g., silvestrol, CR31-B (-), zotatifin) have some limitations such as low permeability, safety issues and an immune modulatory potential [9,10,11]. Studies on the immune modulatory potential, safety profile and bioavailability of pateamines are not available. This indicates a need for studies to clarify whether the immune-suppressive effects, the low permeability and the cytotoxicity observed for rocaglates are due to the inhibition of eIF4A helicase or to drug-specific actions. The aim of this study was to create an immune modulatory profile of pateamines and to analyze selected absorption and safety parameters of this compound class.

Here, we identified the immune modulatory profiles of PatA and DMDA. The impacts of PatA and DMDA on cell viability, cell-type-specific surface markers, released cytokines and energy metabolism during differentiation and polarization/activation of different human immune cells were investigated. Furthermore, bioavailability and safety profiles of both compounds were also determined.

## 2. Results

### 2.1. Pateamines Have Only Marginal Effects on Macrophage Polarization

Recently, we observed immune modulatory effects of the rocaglates silvestrol, zotatifin and CR-31-B (-) [11]. Due to low permeability and strong immune modulatory effects of silvestrol, we investigated further elF4A inhibitors with the intention to identify compounds with a better safety and immune modulatory profile, as well as increased permeability. First, we identified non-cytotoxic concentrations in various immune cell types (B cells, T cells, M1 monocyte-derived macrophages (MdMs), M2 MdMs) for PatA and DMDA. Overall, DMDA seems to be less cytotoxic in comparison with PatA, which is in accordance with previous data [4]. To use non-toxic concentrations for the immune modulatory in vitro experiments, a maximum concentration of 0.5 nM or 5 nM for T/B-cells or macrophages, respectively, were used (Supplementary Materials Figure S1).

**Figure 1:**
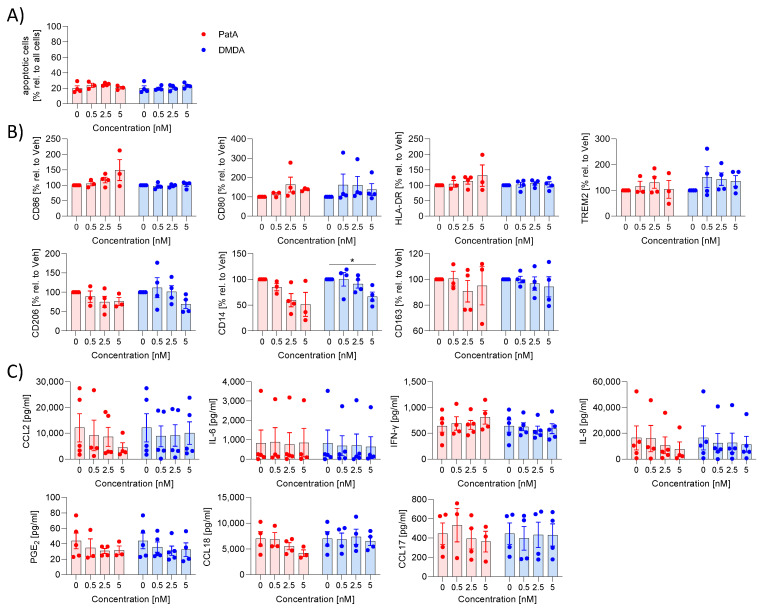
Effect of pateamines on M1 polarization. Human primary monocytes were differentiated to macrophages by the addition of 10 ng/mL GM-CSF in 7 days and polarized by 10 ng/mL IFN-γ to M1 macrophages in the presence or absence of PatA or DMDA for 48 h. (**A**) Apoptosis was determined via zombie staining. (**B**) Surface markers were analyzed via flow cytometry. The MFI values of treated samples were related to vehicle to obtain % values. (**C**) Inflammatory mediators in the supernatant were determined using ELISA or CBA. The experiment was performed with blood from either three (cytokines/surface marker: PatA-treated samples), four (surface marker: DMDA-treated samples and 2.5 nM PatA-treated samples; cytokines: 2.5 nM PatA-treated samples; CCL17: DMDA-treated samples) or five (cytokines: DMDA-treated samples) different donors. For statistical analysis, each compound was analyzed separately via one-way ANOVA with Dunnett’s multiple comparisons test. * *p* < 0.05 indicates significant difference between elF4A inhibitors-treated and vehicle-treated samples. Abb. DMDA, des- methyldes-amino PatA; PatA, pateamine A.

Next, we tested whether the inhibition of elF4A helicase influenced the polarization of MdMs to M1 or M2 MdMs. MdMs were polarized to M1 MdMs by the addition of IFN-γ. Neither PatA nor DMDA induced apoptosis in M1 MdMs (Figure 1A). Several surface markers (CD86, CD80, TREM2, HLA-DR, CD206, CD14, CD163) were analyzed. PatA had no significant effect on surface marker expression. DMDA only reduced CD14 expression (Figure 1B). To investigate whether the elF4A modulators could change the inflammatory environment, the release of cytokines (IL-6, IL-8, CCL2, CCL17, CL18, IFN-γ) and PGE_2_ as an inflammatory mediator were determined. PatA and DMDA did not affect the inflammatory environment (Figure 1C). These data indicate that PatA and DMDA had no influence on the polarization of M1 MdMs.

**Figure 2:**
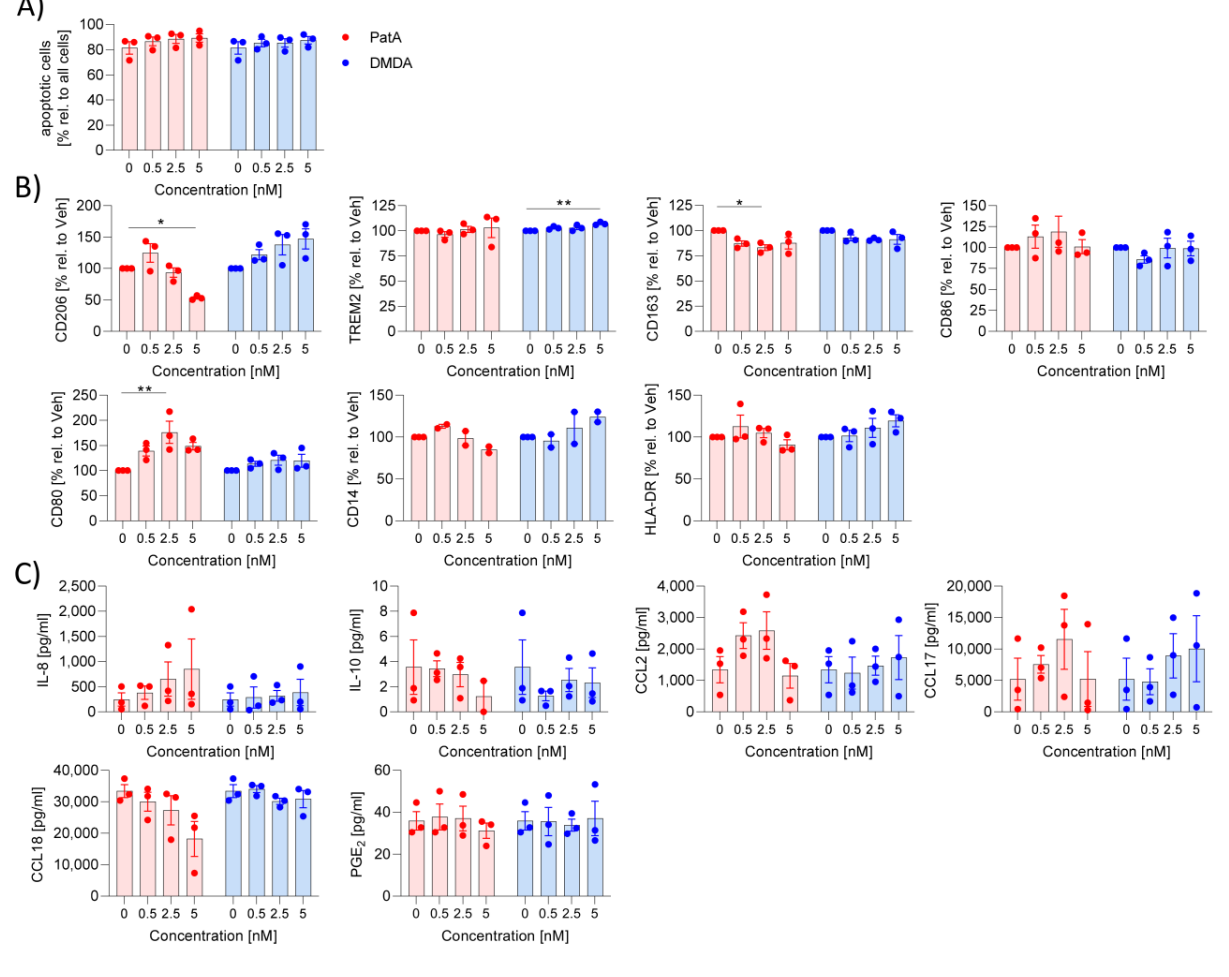
Effect of pateamines on M2 polarization. Human primary monocytes were differentiated to macrophages by the addition of 10 ng/mL M-CSF and polarized by 10 ng/mL IL-4 to M2 macrophages in the presence or absence of PatA or DMDA for 24 h. (**A**) Apoptosis was determined via zombie staining. (**B**) Surface markers were determined via flow cytometry. The MFI values of treated samples were related to vehicle to obtain % values. (**C**) Inflammatory mediators were determined in the supernatant using ELISA or CBA. The experiment was performed with blood from three different donors. Each compound was analyzed separately via one-way ANOVA with Dunnett’s multiple comparisons. * *p* < 0.05 and ** *p* < 0.01 indicate significant difference between elF4A inhibitors-treated and vehicle-treated samples. Abb. DMDA, des-methyl des-amino PatA; PatA, pateamine A.

We also investigated the impact of pateamines on M2 MdMs. MdMs were polarized to M2 MdMs by the addition of IL-4. PatA and DMDA did not induce apoptosis in M2 MdMs (Figure 2A). Treatment of MdMs with IL-4 led to downregulation of CD163 [12], so we used only the CD163^low^ population for analysis. PatA reduced the anti-inflammatory marker CD206 and induced the pro-inflammatory marker CD80 (Figure 2B). An amount of 5 nM DMDA increased the anti-inflammatory surface marker TREM2 (Figure 2B). PatA and DMDA did not affect the inflammatory environment of M2 MdMs (Figure 2C). Overall, PatA and DMDA seemed to have almost no effect on the polarization of macrophages to M1 and M2 MdMs.

### 2.2. PatA and DMDA Did Not Affect T Cell Activation

**Figure 3:**
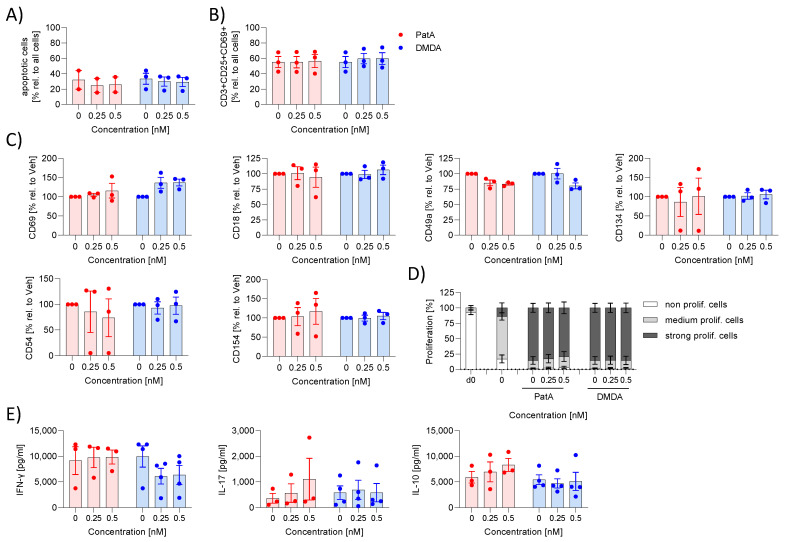
Effect of pateamines on T cell activation. Human primary T cells were activated with anti-CD3/-CD28 and IL-2 in the presence or absence of PatA or DMDA for 5 days. (**A**) Apoptosis was determined via zombie staining. (**B**,**C**) Surface markers were determined via flow cytometry. The MFI values of treated samples were related to vehicle to obtain % values. (**D**) For the proliferation assay, T cells were labeled with the fluorescence dye CTV and the MFI was determined by flow cytometry. Using FlowJo the cells were gated in fractions with high (nonproliferating cells), medium (med. prolif. cells) and low (strong prolif. cells) CTV fluorescence. (**E**) Cytokines were determined in the supernatant by CBA. The experiment was performed with blood from three different donors. Each compound was analyzed separately via one-way ANOVA with Dunnett’s multiple comparisons test. Abb. DMDA, des-methyl des-amino PatA; PatA, pateamine A.

Other elF4A inhibitors (zotatifin, CR-31-B (-)) prevented the activation of T cells [11], therefore, we analyzed whether PatA and DMDA also affected T cell activation. T cells were activated with anti-CD3 and anti-CD28 and the proliferation rate, surface marker expression (CD69, CD154, CD134, CD25, CD18, CD49a, CD54) and cytokine release (IL-17A, IL-10, IFN-γ) were determined. PatA and DMDA showed no apoptotic effects and did not influence the number of activated cells (CD3^+^CD25^+^CD69^+^ cells) (Figure 3A,B). Neither had any effects on surface marker expression, proliferation rate or cytokine release (Figure 3C–E). These data indicate that PatA and DMDA did not affect T cell activation.

### 2.3. PatA Prevented B Cell Activation

**Figure 4:**
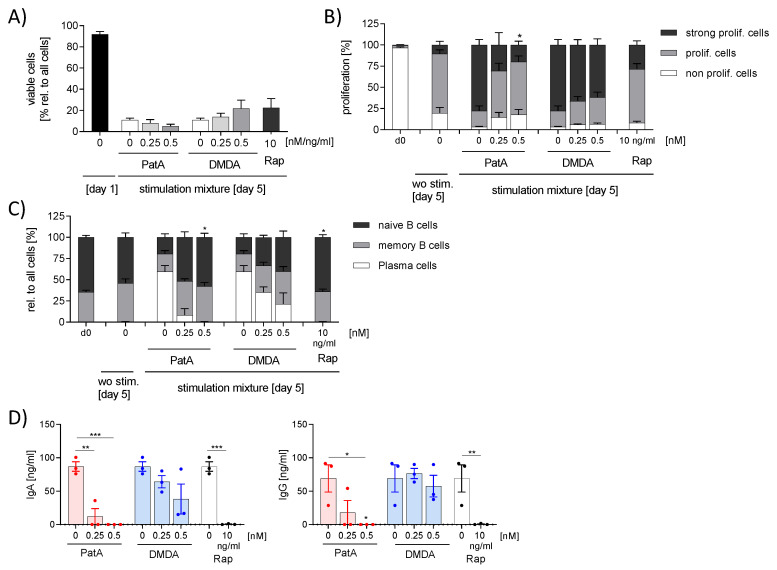
Effect of pateamines on B cell activation. Human primary B cells were activated with 50 ng/mL IL-21, 1 µg/mL sCD40L, 2.5 µg/mL CpG and 5 µg/mL anti-IgM in presence or absence of PatA or DMDA for 5 days. (**A**) Apoptosis was determined by zombie staining. (**B**) For the proliferation assay, B cells were labeled with the fluorescence dye CTV and the MFI was determined via flow cytometry. Using FlowJo, the cells were gated in fractions with high (non-proliferating cells), medium (med. prolif. cells) and low (strong prolif. cells) CTV fluorescence. (**C**) Surface markers were determined via flow cytometry. (**D**) IgG and IgA were analyzed in the supernatant using ELISA. The experiment was performed with blood from three different donors. For statistical analysis, two-way ANOVA (**A**–**C**) or one-way ANOVA (**D**) with Dunnett’s multiple comparisons test was used. * *p* < 0.05, ** *p* < 0.01 and *** *p* < 0.001 indicates a significant difference between elF4A inhibitors-treated and vehicle-treated samples. Abb. DMDA, des-methyl des-amino PatA; PatA, pateamine A.

Recently, we demonstrated that rocaglates (zotatifin, CR-31-B (-), silvestrol) prevented B cell activation [11]. We investigated whether this is a common effect of elF4A inhibitors. B cells were activated with a mixture of IL-21, sCD40L, CpG and anti-IgM for 5 days in the presence or absence of PatA or DMDA. The activation led to proliferation of B cells, formation of plasma cells and the release of immune globulins (Ig)G and IgA. Rapamycin, which inhibits these processes, was used as a control substance. PatA, DMDA and rapamycin did not reduce cell viability (Figure 4A). As expected, rapamycin prevented B cell activation, as shown by the reduced formation of plasma cells, reduced proliferation and release of IgGs (Figure 4B–D). Interestingly, 0.5 nM PatA showed similar effects to those of rapamycin, whereas DMDA did not prevent B cell activation (Figure 4B–D). These data indicate that PatA prevented B cell activation.

### 2.4. PatA Modulated Energy Metabolism in M1 MdMs and B Cells

**Figure 5:**
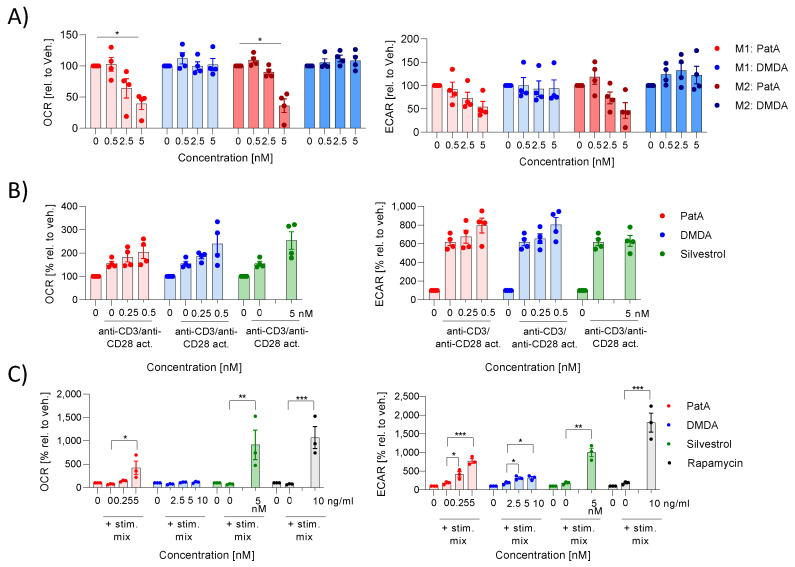
Effect of pateamines on energy metabolism in immune cells. (**A**) Human primary monocytes were differentiated to M1 or M2 macrophages by the addition of 10 ng/mL GM-CSF or 10 ng/mL M-CSF, respectively. Macrophages were polarized by 10 ng/mL IFN-γ or 10 ng/mL IL-4 to M1 or M2 macrophages, respectively, in the presence or absence of PatA or DMDA for 48 h or 24 h, respectively. (**B**) Human primary T cells were cultured with 50 IU/mL IL-2 for 5 days in the presence or absence of PatA, DMDA or silvestrol (5 nM) for 5 days. (**C**) Human primary B cells were cultured with 50 ng/mL IL-21, 1 µg/mL sCD40L, 2.5 µg/mL CpG and 5 µg/mL anti-IgM in the presence or absence of Pat, DMDA, silvestrol (5nM) or rapamycin (10 ng/mL) for 5 days. Oxidative consumption rate (OCR) (left panel) and extracellular acidification rate (ECAR) (right panel) were determined using a Seahorse device. The OCR and ECAR values after 60 min of elF4A inhibitor-treated samples were related to the vehicle-treated samples to obtain fold induction. The experiment was performed with blood from either three (B cells) or four (macrophages, T cells) different donors. For statistical analysis, a two-way ANOVA with Dunnett’s multiple comparisons test was used. * *p* < 0.05, ** *p* < 0.01 and *** *p* < 0.001 indicates significant difference between elF4A modulator-treated and vehicle-treated samples. Abb. DMDA, des-methyl des-amino PatA; PatA, pateamine A.

We have recently shown that M1 macrophages have a higher energy requirement in comparison with M2 macrophages and that they use predominantly oxidative phosphorylation as an energy source to meet their energy requirements. Activated T cells have the highest energy consumption of all tested immune cells (macrophages, B cells) and they use both oxidative phosphorylation and glycolysis. B cells have the lowest energy consumption and also use both energy sources. We also showed, in the previous study, that the energy metabolism of cell types with a high energy requirement (M1 MdMs) is more influenced by rocaglates than is the metabolism of cell types with a low energy requirement (activated monocyte derived dendritic cells) [11]. Silvestrol, in M1 MdMs, reduced glycolysis and oxidative phosphorylation, whereas the energy metabolism in M2 MdMs was not influenced [11]. We investigated whether PatA or DMDA influence the energy metabolism in various immune cell types. The extracellular acidification rate (ECAR) as a marker for glycolysis, and oxygen consumption rate (OCR) as a marker for mitochondrial respiration, were determined. For T and B cells, we also analyzed the effects of silvestrol. In M1 and M2 MdMs, PatA reduced glycolysis and oxidative phosphorylation, whereas DMDA had no effect on energy consumption (Figure 5A). In T cells, PatA, DMDA and silvestrol did not alter the energy metabolism (Figure 5B). Interestingly, in B cells PatA induced oxidative phosphorylation and glycolysis, whereas DMDA induced only glycolysis. Silvestrol also increased glycolysis and oxidative phosphorylation in B cells, which was also the case for the positive control rapamycin (Figure 5C). These data indicate that none of the elF4A inhibitors influenced the energy metabolism of T cells but increased it in B cells. The reason for that could be that in activated T cells the energy metabolism is already high (64.4 ± 4.6 mph/min for ECAR and 102.7 ± 12.7 pmol/min for OCR), whereas in activated B cells the energy metabolism is low (9.8 ± 0.4 mph/min for ECAR and 7.3 ± 0.9 pmol/min for OCR). In M1 and M2 MdMs, the effect on the energy metabolism was drug-specific.

### 2.5. PatA and DMDA Exhibited No Genotoxic or Mutagenic Potential

**Figure 6:**
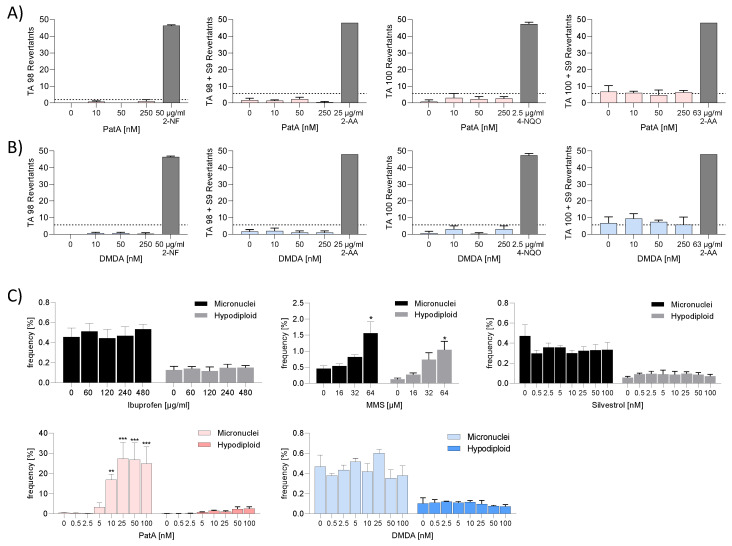
Pateamine A and DMDA revealed no mutagenic potential. The Ames test was conducted with two *Salmonella typhimurium* strains, TA98 and TA100, in the presence or absence of S9 to simulate the metabolic conversions of PatA (**A**) or DMDA (**B**) with liver enzymes. The data were analyzed with the AMES MPF calculation sheet provided by Xenometrix. Fold induction was calculated as the ratio of the mean number of positive wells for the dose concentration divided by the baseline. The baseline (dotted line) was obtained by adding one standard deviation to the mean number of positive wells of the solvent control. Compounds with mutagenic potential are characterized by revertant numbers above the baseline. The experiment was performed once in triplicate. Data are shown ± SD. (**C**) For the micronuclei assay, TK6 cells were incubated with increasing concentrations of PatA, DMDA, silvestrol, ibuprofen (negative control), MMS (positive control) or vehicle for 4 h. Subsequently, the cells were washed and incubated for 44 h. The analyses of micronuclei and hypodiploid cells were achieved via flow cytometry. The experiment was performed three times. For statistical analysis, a two-way ANOVA with Dunnett’s multiple comparisons test was used. * *p* < 0.05, ** *p* < 0.01, *** *p* < 0.001 indicate significant differences between compound and control samples. Abb. 2-AA, 2-aminoanthracene; 2-NF, 2-nitrofluorene; MMS, Methylmethansulfonat; 4-NQO: 4-nitroquinoline-N-oxide; DMDA, des-methyl des-amino PatA; PatA, pateamine A.

To characterize the safety of PatA and DMDA, possible mutagenic and genotoxic effects were assessed. An Ames test was performed to investigate the mutagenic potential of the elF4A inhibitors. PatA and DMDA were tested in two *Salmonella typhimurium* strains, T100 and T98. Liver homogenate S9 was used to simulate metabolic conversion of PatA and DMDA. Both compounds were negative in all Ames mutagenic assays (Figure 6A,B).

The micronuclei assay in human TK6 cells was performed to address the genotoxic potential. The assay allows the determination of micronuclei indicating double-strand breaks and hypodiploids as indications of chromosome loss and thereby, clastogenic and aneugenic compounds can be identified [13]. The positive control, methylmethansulfonat (MMS), led to a significant increase in micronuclei and hypodiploid cells, whereas the negative control, ibuprofen, showed no effects in TK6 cells (Figure 6C). PatA at 10 nM induced clastogenic effects. DMDA induced neither clastogenic nor aneugenic effects. To investigate whether this effect was based on the inhibition of the elF4A or whether it was a drug-specific effect, another elF4A inhibitor (silvestrol) was tested. Silvestrol showed no genotoxic effects (Figure 6C). These data indicate that genotoxicity is a drug-specific rather than a target-specific effect of PatA.

### 2.6. Low Permeability of PatA and DMDA in the Caco-2 and Calu-3 Cell Barrier Assay

**Figure 7:**
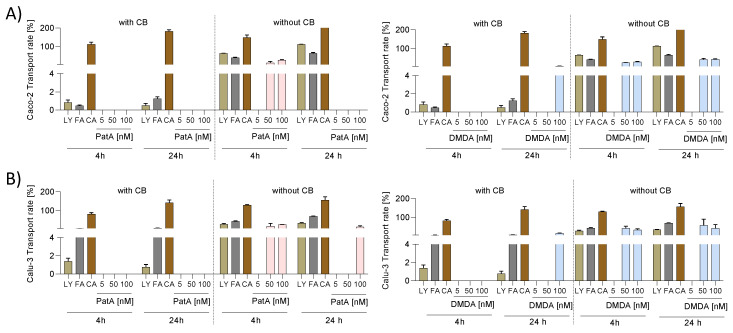
Permeability of PatA and DMDA. (**A**,**B**) To determine the transport rate of PatA and DMDA, Caco-2 (**A**) or Calu-3 (**B**) cell barriers were incubated with various concentrations of PatA and DMDA. The permeability controls were 200 µM carbamazepine (high permeability), 200 µM famotidine (low permeability) and 200 µM lucifer yellow (low permeability) incubated for 4 h or 24 h. As a control, trans-well filters without cell barriers were used. The concentration of the compounds in the apical and basolateral medium were determined using LC-MS/MS or via fluorescence detection. To obtain the transport rate, the drug amount in the basolateral compartment at 4 h or 24 h was related to the drug amount in the apical compartment (0 h). The experiment was performed in biological triplicates (Calu-3) or quadruplicates (Caco-2). Data are expressed as mean ± SEM. Abb. CA, carbamazepine; DMDA, des-methyl des-amino PatA; FA, famotidine; LY, lucifer yellow; PatA, pateamine A.

Besides oral application, inhalation could also be a possible administration route for eIF4A inhibitors. Therefore, we investigated permeability in cell barrier assays composed of a lung cancer cell line (Calu-3) or a colon cancer cell line (Caco-2). For this purpose, the transport rate for both compounds across Caco-2 and Calu-3 cell barriers was determined. The compounds carbamazepine and famotidine/lucifer yellow were used as high and low permeability controls, according to FDA guidelines for bioavailability [14]. The Caco-2 or Calu-3 cell barrier was generated on a porous membrane and covered with PatA (5, 50, 100 nM), DMDA (5, 50, 100 nM), 200 µM carbamazepine, 200 µM famotidine or 200 µM lucifer yellow for 4 and 24 h. As a control, porous membranes without cell barriers were incubated with the different compounds. As expected, the transport rate of carbamazepine was high, whereas famotidine and lucifer yellow revealed a low permeability (Figure 7A,B). Astonishingly at 100 nM, PatA only revealed a transport rate of about 28% without a cell barrier (i.e., just from the apical to the basolateral compartments in the absence of cells) and that only after 4 h, indicating that PatA is probably unstable in the media under these assay conditions. With cell barriers, no permeability was detected for PatA (Figure 7A,B). Without a cell barrier for 100 nM DMDA, transport rates of about 28 and 41% after 4 and 24 h, respectively, were observed. With Caco-2 or Calu-3 cell barriers, transport rates of about 6 and 14%, respectively, after 24 h was calculated for 100 nM DMDA (Figure 7A,B). In summary, PatA was characterized by zero permeability and seems to be unstable while DMDA exhibited low permeability.

## 3. Discussion

We studied the effects of elF4A inhibitors, DMDA and PatA on immune cell function, bioavailability and in vitro safety. DMDA exhibited almost no immune modulatory effects within the tested concentration range of 0.5–5 nM. PatA; on the other hand, it reduced B cell activation, while macrophage and T cell function remained unaffected. Overall, PatA appears to downregulate the immune response. However, PatA may induce genotoxicity and has zero permeability. In contrast, DMDA showed a good safety profile and low permeability.

Recently, we analyzed the immune modulatory potential of the rocaglates CR-31-B (-), silvestrol and zotatifin [11]. Pateamines, as well as rocaglates, have the same mode of action in inhibiting eIF4A. They clamp RNA substrates onto the surface of the helicase, thereby blocking the conformational change from a closed to a half-open conformation, which is required for the enzymatic activity of DEAD-box helicases to unwind RNA double-strands [15]. A comparison of various elF4A inhibitors (PatA, DMDA, silvestrol, CR-31-B (-), zotatifin) for their effects on the release of inflammatory markers from immune cells revealed that all elF4A modulators, except DMDA, affect immune cells (Table 1). The immune modulatory effect of elF4A modulators was also observed by others. Zhao et al. demonstrated that zotatifin inhibits IFN-γ production and repolarization of tumor-associated macrophages to M1 macrophages [16]. PatA and hippuristanol, both elF4A inhibitors, inhibit cytokine production via elF4A and STAT 3 [17]. Potentially, the immune modulatory effects may be linked to the antiviral activity of eIF4A inhibitors. In this respect, to achieve comparable antiviral activity between DMDA and PatA, a 10-fold higher concentration of DMDA (approximately 30 nM) may be required to induce immune modulatory effects [4].

The reduction in the release of immunoglobulins (IgA, IgG) from B cells by elF4A inhibitors certainly appears to be elF4A dependent, as 4 out of 5 inhibitors exhibited this effect. Moreover, in M2 and M1 MdMs, a target-specific effect was observed, as the inhibition of elF4A by 2–3 out of 5 inhibitors led to a reduced release of chemokines, potentially impairing the recruitment of further immune cells. The effect on T cells seems to be drug-specific, with three different outcomes: (1) PatA and DMDA did not affect T cells; (2) silvestrol increased cytokine release; and (3) CR-31-B (-) and zotatifin (both synthetic rocaglates without the dioxan moiety of silvestrol) reduced cytokine release, as shown previously for PatA by others [18].

The inhibition of elF4A in M1 MdMs may lead to a reduced immune response in case of viral infections, as reported for IFN-γ with zotatifin [16], as macrophages are key cells of the innate immune system and the first line of defense. Macrophages release chemokines and cytokines to recruit and stimulate immune cells of the adaptive immune system, such as B cells and T cells. Consequently, the reduced release of chemokines (IL-8, CCL2) in macrophages by elF4A inhibition can hinder the recruitment of additional immune cells and may weaken the host’s immune response. B cells play a unique part by producing antibodies that can neutralize and clear viral particles before viruses enter cells. Four of five elF4A inhibitors, in our experiments, prevented the production of antibodies (IgG, IgA), potentially further reducing the host adaptive immune response. In summary, both macrophages and B cells mediate important steps in viral clearance and inhibiting their function through elF4A inhibition could counteract the antiviral effects of elF4A inhibitors and weaken the overall antiviral response.

In the case of viral infections, macrophages are often exploited by viruses for their replication and propagation. Infections trigger significant metabolic reprogramming within macrophages, involving alterations in basic pathways such as the Krebs cycle, glycolysis and the pentose phosphate pathway [19]. Glycolysis plays a crucial role in macrophage phagocytic action and its inhibition with 2-DG can reduce phagocytosis [20]. Since PatA reduces glycolysis in M1 macrophages, it potentially also impairs the phagocytic potential of macrophages.

The elF4A inhibitors are under investigation in the field of cancer. Effects on B cells may be beneficial or harmful for tumor cells. B cells use fundamentally unique mechanisms to distinguish self from non-self, making them natural partners for tumor surveillance and control [21]. Tumor-infiltrating B cells secrete anti-tumor antibodies and tumor cells coated with IgG or IgA have been observed in association with B cells in renal cell carcinoma, soft-tissue sarcoma, breast cancer and endometrial cancer [22,23,24,25]. Since all investigated elF4A inhibitors except DMDA inhibit B cell activation and the release of IgG and IgA, this immune-suppressive effect can potentially reduce the anti-tumorigenic potential of elF4A inhibitors. Moreover, B cells can be harmful to tumor cells via antibody dependent cell cytotoxicity (ADCC). But they can also affect tumor growth by fueling chronic inflammation, angiogenesis, or immunosuppression via immune complex formation or complement activation [26]. Exploring the targeted delivery of eIF4A inhibitors could help mitigate unwanted effects on the immune system and enhance bioavailability as a therapeutic option.

Tumor-associated macrophages (TAMs) are generally characterized as an M2-like macrophage phenotype within the tumor microenvironment. TAMs often accelerate the progression of untreated cancer and negatively affect the efficacy of anticancer drugs, including checkpoint inhibitor immunotherapies. TAMs secrete numerous immunosuppressive cytokines and factors, including IL-10, TGF-β and ROS, which induce CD8^+^ T cell exhaustion and dysfunction [27]. Efforts are underway to either deplete M2 cells or convert the M2 phenotype into M1 macrophages in most tumors [27,28]. The elF4A inhibitors showed only minor effects on M2 macrophages. However, for silvestrol we could show that it potentially strengthens the M2 phenotype by reducing expression of CD206, TREM2 and decreasing the release of pro-inflammatory IL-8 and CCL2, thereby possibly weakening the host’s immune defense against cancer by exhausting CD8^+^ T cells [10].

The inhibition of RNA-bound eIF4A by rocaglates affects only about 300 mRNAs in our cells primarily involved in regulating proliferation [29]. Due to differences in the binding modes of pateamines compared with rocaglates, we cannot rule out additional off-target effects for pateamines at this time. However, rocaglates have an acceptable safety profile (showed no off-target effects in a safety screen panel) and ribosome profiling with DMDA-PatA shows a similar profile to rocaglates [7]. The relevance of pateamines and also rocaglates as antivirals may be limited to local applications, such as aerosol-based delivery into the lungs, to avoid unwanted effects on antibody production in B cells.

In conclusion, inhibiting elF4A appears, in general, to impair the function of macrophages and B cells, while its effect on T cells seems to be individually drug-specific. These findings suggest that the immune-suppressive effect of elF4A inhibitors on B cells may weaken the immune response against viral infections and the defense against tumor cells.

## 4. Materials and Methods

### 4.1. Cells and Reagents

Used materials are listed in Table 2. Human primary monocytes and monocyte-derived macrophages (MdMs) were cultured in RPMI 1640-Glutamax medium supplemented with 1% penicillin/streptomycin and 10% FCS. Human primary T cells were cultured in RPMI 1640 medium supplemented with 2 mM glutamine, 1% penicillin/streptomycin and 10% FCS. Human primary B cells were cultured in RPMI 1640 medium supplemented with 2 mM glutamine, 0.05 mM ß-mercaptoethanol, 1% penicillin/streptomycin and 10% FCS. Caco-2 cells were cultured in EMEM supplemented with 10% FCS, L-glutamine and non-essential amino acids. Calu-3 cells were cultured in MEM with 10% FCS and 1x non-essential amino acids. TK6 cells were cultured in RPMI 16040 medium supplemented with 2 mM glutamine, 10% horse serum, 1 mM sodium pyruvate and 1% penicillin/streptomycin. All cells were cultured at 37 °C in 5% CO_2_ atmosphere. Buffy coats from healthy donors were obtained freshly from the DRK-Blutspendedienst (Frankfurt am Main, Germany). Silvestrol (purchased from the Sarawak Biodiversity Centre, Kuching, Borneo at a purity of > 99%) was dissolved in DMSO and further diluted in medium (c_stock_ = 6 mM, maximal DMSO concentration during experiments 0.000083% *v*/*v*). Pateamine A and des-methyl des-amino pateamine A (DMDA) were a gift from Prof. Alois Fürstner (Max Planck Institute, Mülheim, Germany) and dissolved in DMSO at a concentration of 100 µM and stored at –20 °C [30,31].

### 4.2. Isolation of Human CD14^+^ Cells, CD4^+^ Cells and B Cells

Human CD14^+^ cells, CD4^+^ cells and B cells were isolated as described previously [11]. Briefly, human peripheral blood mononuclear cells (PBMCs) were isolated from fresh buffy coats by density gradient centrifugation as previously described [10]. CD4^+^ and CD14^+^ cells were isolated with human CD4 or CD14 MicroBeads (Miltenyi Biotec, Bergisch Gladbach, Germany) according to the protocol of the supplier. Defined amounts of cells were dissolved in 0.5% BSA/2 mM EDTA/PBS and incubated with 25% (*v*/*v*) human CD14 or CD4 MicroBeads for 15 min at 4 °C. After incubation, the magnetic labeled cells were separated from unlabeled cells via magnetic cell separation (MACS) with LS columns. The purity of monocytes and T cells after isolation was about 86% and 95%, respectively.

B cells were isolated with the B cell isolation kit (Miltenyi Biotec, Bergisch Gladbach, Germany) according to the protocol of the supplier. Briefly, defined amounts of cells were dissolved in 0.5% BSA/2 mM EDTA/PBS and incubated with 25% (*v*/*v*) biotin antibody cocktail for 5 min at 4 °C. The anti-biotin MicroBeads were added and incubated for 10 min at 4 °C. After incubation the magnetic labeled cells were separated from unlabeled cells via magnetic cell separation (MACS) with LS columns. The unlabeled cells (enriched B cells) were collected. The cell counts were determined using MACSQuant^®^ Analyzer 10 and MACSQuantify^TM^ Software 2.13.3. The purity of B cells after isolation was about 95%.

### 4.3. Differentiation of Human Macrophage

The differentiation of human monocytes to macrophages (MdMs) was performed as previously described [10]. Briefly, 0.5 × 10^6^ CD14^+^ cells/well were pretreated with different concentrations of PatA and DMDA or vehicle (DMSO) for 30 min. After pre-incubation, differentiation was initiated by the addition of 10 ng/mL GM-CSF for M1 MdMs or 50 ng/mL M-CSF for M2 MdMs and incubated for 7 days.

### 4.4. Polarization of Human Macrophages

The polarization of MdMs to M1 or M2 MdMs was performed as previously described [10]. M1 and M2 MdM polarization was conducted in separate experiments. Briefly, M1 MdMs were polarized in presence of PatA (0.5, 2.5, 5 nM), DMDA (0.5, 2.5, 5 nM) or vehicle (DMSO) with 20 ng/mL human IFN-γ for 48 h. M2 MdMs were polarized in presence of PatA (0.5, 2.5, 5 nM), DMDA (0.5, 2.5, 5 nM) or vehicle (DMSO) with 10 ng/mL human IL-4 for 24 h.

### 4.5. Activation of T Cells and B Cells

T cells were activated as previously described [11]. Briefly, 1.75 × 10^5^ T cells (stained with 0.2 µM CellTrace^TM^ violet (CTV) (ThermoFisher Scientific, Schwerte, Germany)) per well were pretreated with PatA (0.25, 0.5 nM), DMDA (0.25, 0.5 nM) or vehicle (DMSO) for 30 min in RPMI 1640 medium supplemented with 2 mM glutamine, 50 Units/mL IL-2, 1% penicillin/streptomycin and 10% FCS. Afterwards, they were seeded in CD3-coated 96-well plates and stimulated with 2 µg/mL CD28 for 5 days. CTV staining was performed according to the protocol of supplier.

B cells were activated as described by Khoenkhoen et al. [32]. Briefly, 1.25 × 10^5^ B cells (stained with 0.25 µM CTV) per well were seeded in a 48-well plate and pretreated with PatA (0.25, 0.5 nM), DMDA (0.25, 0.5 nM) or vehicle (DMSO) for 30 min and then stimulated with 5 μg/mL unconjugated goat anti-human IgM F (ab’)2 fragments, 2.5 μg/mL CpG, 1 μg/mL sCD40L and 50 ng/mL recombinant human IL-21 for 5 days.

### 4.6. Surface Marker Detection and Proliferation Assay by Flow Cytometry

Surface marker analysis was performed as described previously [11]. Briefly, 1.5–2 × 10^5^ cells of each sample were blocked with human FcR Blocking Reagent (15 min, 4 °C). A Zombie Aqua™ Fixable Viability Kit (1:500 dilution, BioLegend, San Diego, CA, USA) was used according to the manufactory protocol to discriminate living and dead cells. Afterwards, samples were stained with a mixture of different surface marker antibodies (15 min, 4 °C) and measured with MACSQuant^®^ Analyzer 10 flow cytometer. For M1-/M2-MdMs anti-CD86, anti-CD14, anti-HLA-DR, anti-CD206, anti-CD80, anti-TREM2 and anti-CD163 were used. For T cells, anti-CD69, anti-CD54, anti-CD49a, anti-CD18, anti-CD134, anti-CD154, anti-CD3 and anti-CD25 were analyzed. For B cells, anti-CD27, anti-CD19 and anti-CD38 were applied. For T cell and B cell proliferation assay, freshly isolated T and B cells were stained with CTV and detected using flow cytometry. Naïve B cells were characterized as CD19^+^CD27^low^CD38^med^, memory B cells as CD19^+^CD27^med^ CD38^low^ and plasma cells as CD27^+^CD38^+^ cells. M1 MdMs and M2 MdMs cell population was gated using FSC/SSC channel. T cells were gated as CD3^+^CD25^+^ positive cells. The geometric mean of the fluorescent intensity was calculated using FlowJo software v10 (Treestar, Ashland, TN, USA). Fold induction of surface marker expression was calculated using the DMSO-treated cells as the control.

### 4.7. Cytokine, Immunoglobulin and PGE_2_ Detection by Cytometric Bead Array or ELISA

For determination of cytokine concentrations in the supernatant of humane immune cells, cytometric bead array (BD Biosciences, Heidelberg, Germany) for IL-10, IL-17, IL-8, IL-6, IFN-γ and CCL2 was used. For the detection of CCL18, CCL17, IgG, IgA and PGE_2_, an ELISA was performed. The cytometric bead arrays and the ELISAs were performed according to the protocol of the supplier.

### 4.8. Determination of Energy Metabolism

The determination of the energy metabolism was performed as described previously [10]. Briefly, for the analysis of the extracellular acidification rate (ECAR) and the oxygen consumption rate (OCR) for human B cells, Tcells, M1 MdMs and M2 MdMs, the Seahorse XFe96 FluxPak (Agilent, Waldbronn, Germany) was used as recommended by the manufacturer. MdMs were polarized to M1 or M2 MdMs in the presence of PatA (0.5, 2.5, 5 nM), DMDA (0.5, 2.5, 5 nM) or vehicle (DMSO) for 48 h or 24 h, respectively. T cells were activated in the presence of PatA (0.25, 0.5 nM), DMDA (0.25, 0.5 nM), silvestrol or DMSO. B cells were activated in the presence of PatA (0.25, 0.5 nM), DMDA (0.25, 0.5 nM), silvestrol (5 nM) or rapamycin (10 ng/mL positive control). Cells were washed with Seahorse XF RPMI medium pH 7.4 (Agilent, Waldbronn, Germany) and OCR and ECAR were measured as octuplicates for a period of 160 min using the Seahorse XFe96 Analyzer (Agilent, Waldbronn, Germany) and analyzed by Wave Software 2.6.3.8 (Agilent, Waldbronn, Germany). The relative values were calculated by relating the OCR or ECAR values obtained after 50 min to the values of the vehicle control (not stimulated).

### 4.9. Ames Test

The Ames MPF 98/100 test (Xenometrix, Allschwil, Switzerland) was performed as recommended by the supplier and recently published [33]. Briefly, two bacteria strains incapable of producing histidine (Salmonella typhimurium strains TA98 and TA100) were used. Liver homogenate S9 was applied to simulate the metabolic conversions of PatA and DMDA. After exposure to increasing concentrations of PatA (0, 10, 50, 250 nM), DMDA (0, 10, 50, 250 nM) or with positive controls (2 μg/mL for 2-NF (TA98), 2.5 μg/mL for 4-NQO (TA100), 63 μg/mL (TA100 with SF9) and 25 µg/mL (TA98 with SF9) for 2-AA), the cultures were diluted in pH indicator medium lacking histidine and incubated for two days. Cells that had undergone reversion to amino acid prototrophy grew into colonies and bacterial metabolism reduced the pH of the medium, changing the color of that well. Revertant colony numbers of each well were counted and related to the negative control (DMSO). The experiment was conducted once in triplicate. The Ames MPF calculation sheet provided by Xenometrix was used for data analysis. Fold induction over the baseline was calculated as the ratio of the mean number of positive wells to the baseline. The baseline was obtained by adding one standard deviation to the mean number of positive wells of the solvent control. Compounds with mutagenic potential were characterized by revertant numbers above the baseline.

### 4.10. Micronuclei Assay

The In-Vitro Micronuclei Plus assay (Becton Dickinson, Heidelberg, Germany) was performed as described by the supplier. Briefly, 2 × 10^4^ TK6 cells were incubated with increasing concentrations of PatA, DMDA and silvestrol for 4 h. The cells were washed with PBS and incubated in fresh medium (without test substance) for 44 h. The cells were centrifuged (300× *g*) for 5 min, the supernatant was collected and placed on ice for 20 min. Then, 50 µL of Complete Nucleic Acid Dye A Solution was added. The plate was placed on ice and exposed to visible light for 30 min. The Nucleic Acid Dye A solution was removed and the cells were washed with 0.15 mL of cold 1x Buffer Solution. An amount of 100 µL Complete Lysis Solution 1 was added, followed by gently mixing of the plate for 5 s and incubation of 1 h in the dark at 37 °C. Then, 100 µL Complete Lysis Solution 2 was added and incubated for 30 min in the dark at room temperature. The cells were analyzed via flow cytometry with the MACSQuant 10 (Miltenyi GmbH, Bergisch Gladbach, Germany).

### 4.11. Transport Assay

The transport assay was achieved as described previously [33]. Briefly, 1 × 10^5^ CaCo-2 or 5 × 10^4^ Calu-3 cells in 300 µL medium were seeded on 24-well ThinCerts (pre-coated with FCS for 60 min) and in the lower compartment, 1 mL culture medium per well was added. The Caco-2 cell barrier was generated over 20 days and the Calu-3 barrier was generated over 14 days. DMDA (0 nM, 5 nM, 50 nM, 100 nM), PatA (0 nM, 5 nM, 50 nM, 100 nM), 200 µM famotidine, 200 µM lucifer yellow or 200 µM carbamazepine diluted in medium was added to the apical compartment and incubated for 4 h or 24 h. The medium from the basolateral and apical compartment was collected and stored at −20 °C and analyzed using LC-MS/MS. To obtain the transport rate, the drug amount in the basolateral compartment at 4 h or 24 h was related to the drug amount in the apical compartment (0 h).

### 4.12. Determination of DMDA, PatA, Carbamazepin, Famotidine via LC-MS/MS

The detailed method for the analysis of DMDA, PatA, carbamazepine and famotidine using LC-MS/MS is described in the Supplementary Materials.

### 4.13. Statistics

Results are presented as means ± standard errors (SEM) unless otherwise described in the legend. For all calculations and creation of graphs, GraphPad Prism 9 was used and *p* < 0.05 was considered as the threshold for significance. Applied statistical analysis are denoted in the figure legends. In every test, the elf4A inhibitor treatment was compared to vehicle.

## Figures and Tables

**Table 1:** Summary of the effects of elF4A modulators on the release of inflammatory markers.

	M1 MdM	M2 MdM	T Cells	B Cells
PatA	No effect	**No effect**	No effect	**IgA↓**, **IgG↓**
DMDA	No effect	No effect	No effect	No effect
Silvestrol [11]	IL-6↓, **IL-8↓**, CCL2↓, TNF-α↑	IL-8↓, **CCL2↓**,	IL-10↑, IFN-γ↑	**IgA↓**, **IgG↓**
CR31-B (-) [11]	IL-6↓, **IL-8↓**, CCL2↓, CCL18↓	TNF-α↑, **CCL2↓**, CCL17↓, **CCL18↓**,	IL-10↓, IL-17↓, IFN-γ↓	**IgA↓**, **IgG↓**
Zotatifin [11]	No effect	IL-10↓, **CCL18↓**	IL-10↓, IL-17↓, IFN-γ↓	**IgA↓**, **IgG↓**

**Bold:** At least 3 elF4A inhibitors affect these inflammatory markers. ↑ upregulation; ↓ downregulation.

**Table 2:** List of materials.

Cells/Chemicals/Reagents/Kits	Supplier
Accutase^®^	Merck (Darmstadt, Germany)
Antibodies (except CD54)	Miltenyi Biotec (Bergisch Gladbach, Germany)
B cell isolation kit II	Miltenyi Biotec (Bergisch Gladbach, Germany)
BioColl Trennlösung^®^	Bio&Sell (Feucht, Germany)
Bovine serum albumin (BSA)	Miltenyi Biotec (Bergisch Gladbach, Germany)
Caco-2 cells	Sigma Aldrich (Schnelldorf, Germany)
Calu-3 cells	ATCC (Manassas, USA)
CCL17 ELISA	Biolegend (Fell, Germany)
CCL18 ELISA	BosterBio (Pleasanton, CA, USA)
CD4 MicroBeads	Miltenyi Biotec (Bergisch Gladbach, Germany)
CD14 MicroBeads	Miltenyi Biotec (Bergisch Gladbach, Germany)
sCD40L	Peprotech (Hamburg, Germany)
Anti-CD54	Biolegend (Fell, Germany)
Cell Trace violet	Thermo Fisher Scientific (Oberhausen, Germany)
CpG ODN 2395	InvivoGen (Toulouse, France)
Cytometric bead array	BD Biosciences (Heidelberg, Germany)
EDTA	Sigma-Aldrich (Schnelldorf, Germany)
EMEM medium	Sigma Aldrich (Schnelldorf, Germany)
FcR Blocking Reagent	Miltenyi Biotec (Bergisch Gladbach, Germany)
Glutamine	Gibco (Thermo Fisher Scientific, Oberhausen, Germany)
Granulocyte macrophage colony-stimulating factor (GM-CSF), human	Miltenyi Biotec (Bergisch Gladbach, Germany)
IgG ELISA	Thermo Fisher Scientific (Oberhausen, Germany)
IgA ELISA	Thermo Fisher Scientific (Oberhausen, Germany)
IL-4, human	Miltenyi Biotec (Bergisch Gladbach, Germany)
IL-21 ELISA	Biolegend (Fell, Germany)
Macrophage colony-stimulating factor (M-CSF), human	Miltenyi Biotec (Bergisch Gladbach, Germany)
MEM medium	Gibco (Thermo Fisher Scientific, Oberhausen, Germany)
Non-essential amino acids	M7145, Sigma Aldrich (Schnelldorf, Germany)
Orangu™	Cell guidance systems (Cambridge, UK)
PGE_2_ ELISA	Enzo Life Sciences (Lörrach, Germany)
Rapamycin	Biomol GmbH (Hamburg, Germany)
RPMI 1640-Glutamax	Gibco (Thermo Fisher Scientific, Oberhausen, Germany)
RPMI 1640 medium	Gibco (Thermo Fisher Scientific, Oberhausen, Germany)
TK6 cells	Sigma Aldrich (Schnelldorf, Germany)
Unconjugated goat anti-human IgM F(ab‘)2 fragments	Biomol GmbH (Hamburg, Germany)

## Data Availability

The data that support the findings of this study are available from the corresponding author upon reasonable request.

## Supplementary Materials

The following supporting information can be downloaded at: https://www.mdpi.com/article/10.3390/ijms252111430/s1.

## Abbreviations

ANOVA: analysis of variance; BSA, bovine serum albumin; CCL, CC-chemokine ligand; CTV, CellTrace^TM^ violet; ECAR, extracellular acidification rate; eIF4a, eukaryotic initiation factor 4a; FCS, fetal calf serum; GM-CSF, granulocytes macrophage colony-stimulating factor; IFN-γ, interferon gamma; IL, interleukin; MACS, magnetic activated cell sorting; MdM, monocyte-derived macrophages; OCR, oxygen consumption rate; PBMCs, human peripheral blood mononuclear cells; PBS, phosphate-buffered saline; RAP, rapamycin; SEM, standard error of the mean; TNF-α, tumor necrosis factor alpha.
