# Supplementary material for: Immune Modulatory Profile of the Pateamines PatA and Des-Methyl Des-Amino PatA
Source: Int J Mol Sci. 2024 Oct 24;25(21):11430. doi: 10.3390/ijms252111430 (PMC11546719; doi:10.3390/ijms252111430)
Supplement: Supplementary file 1 [file ijms-25-11430-s001.zip › ijms-3236943-supplementary.pdf]

Supplementary Materials

Immune Modulatory Profile of the Pateamines PatA and Des-Methyl Des-Amino PatA

Susanne Schiffmann [1,2,*], Marina Henke [1], Sophie Brünner [1], Alexandre Bennett [1,2], Yassin Yagubi [1,2], Francesca Magari [3], Michael J. Parnham [1,4] and Arnold Grünweller [3]

1 Fraunhofer Institute for Translational Medicine and Pharmacology ITMP, Theodor-Stern-Kai 7, 60596 Frankfurt am Main, Germany; mike.j.parnham@gmail.com (M.J.P.)
2 Faculty of Medicine, Institute of Clinical Pharmacology, Goethe University Frankfurt, Theodor-Stern-Kai 7, 60590 Frankfurt am Main, Germany
3 Institute of Pharmaceutical Chemistry, Philipps-University Marburg, Marbacher Weg 6, 35032 Marburg, Germany; magari@staff.uni-marburg.de (F.M.)
4 EpiEndo Pharmaceuticals ehf, Bjargargata 1, 102 Reykjavik, Iceland
* Correspondence: susanne.schiffmann@itmp.fraunhofer.de; Tel.: +49-69-8700-25060; Fax: +49-69-8700-10000

Method

Determination of DMDA, PatA, carbamazepin, famotidine via LC-MS/MS

The analysis of carbamazepine was described previously [1]. Briefly, medium was mixed with the internal standard (IS) solution (carbamazepine-d10, 5 ng/ml in methanol) and methanol. For calibration standards and quality control samples, 50 µl blank medium were spiked with 20 µl of standard working solutions and processed as for the samples. After vortexing and centrifugation, 100 µl of the liquid phase were transferred to glass vials. The analysis was performed using an Agilent 1290 Infinity LC system (Agilent, Waldbronn, Germany) coupled to a hybrid triple quadrupole linear ion trap mass spectrometer QTRAP 6500+ (Sciex, Darmstadt, Germany) equipped with a Turbo-V-source operating in positive electrospray ionization mode. The chromatographic separation was carried out using a Zorbax C8 Eclipse Plus RRHD column (50x 2.1 mm, 1.8 µm particle size; Agilent, Santa Clara, USA), maintained at 40 °C. Mobile phase A was 0.1% formic acid + 10 mM ammonium formate and mobile phase B was acetonitrile + 0.0025% formic acid. A gradient program was employed at a flow rate of 300 µl/min, as follows: initial 40% B to 0.3 min, increasing to 70% B from 0.3 to 1.0 min, and to 100% B from 1.0 to 1.8 min, with a hold time of 0.2 min. Re-equilibration was performed by reducing to 40% B from 2.0 to 2.5 min, and then holding at 40% B until 4 min.

For the analysis of famotidine, 40 µl sample were spiked with 20 µl methanol, 20 µl IS (famotidine-d4, 100 ng/mL in methanol), 280 µl H2O and 40 µl methanol. For calibration standards and quality control samples, 40 µl blank medium were spiked with 20 µl standard working solution, 20 µl IS, 280 µl H2O and 40 µl methanol. After vortexing and centrifugation, the supernatant was transferred to glass vials. The analysis was performed with an Agilent 1290 Infinity LC system (Agilent, Waldbronn, Germany) coupled to a hybrid triple quadrupole linear ion trap mass spectrometer QTRAP 6500+ (Sciex, Darmstadt, Germany) equipped with a Turbo-V-source operating in positive electrospray ionization mode. The chromatographic separation was carried out using a Synergi Hydro RP column (150x 2mm, 4 µm; Phenomenex, Aschaffenburg, Deutschland), maintained at 50 °C. Mobile Phase A was 10 mM ammonium acetate and mobile Phase B was acetonitrile. A gradient program was employed at a flow rate of 400 µl/min, as follows: initial 10% B to 0.5 min, increasing to 70% B from 0.5 to 1.5 min, with a hold time of 2.0 min. Re-equilibration was performed by reducing to 10% B from 3.5 to 3.6 min, and then holding at 10% B until 5.5 min.

For the analysis of PatA and DMDA, to 100 µl sample were added 20 µl acetonitrile, 20 µl IS (telmisartan, 2 ng/mL in acetonitrile), and 600 µl of chloroform/methanol/hydrochloric acid (83:15:2; v/v/v). For calibration standards and quality control samples, 100 µl blank medium were spiked with 20 µl standard working solution, 20 µl IS and 600 µl chloroform/methanol/hydrochloric acid (83:15:2; v/v/v). After vortexing and centrifugation, the lower phase was transferred to glass vials, evaporated to dryness at 45 °C under

nitrogen, and the residue dissolved in 50 µl methanol. The analysis was performed with an Agilent 1290 Infinity LC system (Agilent, Waldbronn, Germany) coupled to a hybrid triple quadrupole linear ion trap mass spectrometer QTRAP 6500+ (Sciex, Darmstadt, Germany) equipped with a Turbo-V-source operating in positive electrospray ionization mode. The chromatographic separation was carried out using an Acquity UPLC BEH C18 column (100x 2.1 mm, 1.7 µm; Waters, Eschborn, Deutschland), maintained at 40 °C. Mobile Phase A was 0.1% formic acid with 20 mM ammonium formate and mobile Phase B was methanol with 0.1% formic acid with 20 mM ammonium formate. A gradient program was employed at a flow rate of 400 µl/min, as follows: initial 20% B to 0.5 min, increasing to 90% B from 0.5 to 4.5 min, with a hold time of 1.0 min. Re-equilibration was performed by reducing to 20% B from 5.5 to 5.6 min, and then holding at 20% B until 7.0 min.

For analysis and quantification of all compounds, Analyst Software 1.7.1 and Multiquant Software 3.0.3 (both Sciex, Darmstadt, Germany) were used. The precursor-to-product ion transitions used for quantification were: m/z 237.1 → m/z 194.1 for carbamazepine, m/z 247.1 → m/z 204.1 for carbamazepine-d10, m/z 338.1 → m/z 259.1 for famotidine, m/z 342.1 → m/z 263.1 for famotidine-d4, m/z 556.3 → m/z 511.2 for PatA, m/z 527.2 → m/z 482.2 for DMDA, and m/z 237.1 → m/z 194.1 for telmisartan. Calibration curves were constructed using linear regression with 1/x weighting. Variations in accuracy were less than 15% over the whole range of calibration, except for the lowest limit of quantification, where a variation in accuracy of 20% was accepted.

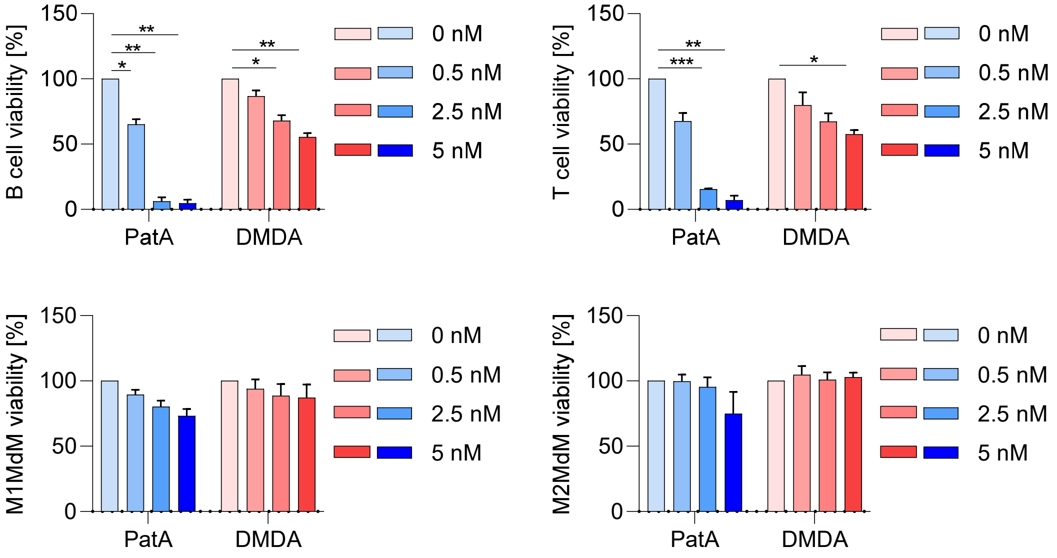

Supplementary Figure S1. Effect of pateamines on immune cell viability. T cells, B cells, M1 MdMs and M2 MdMs were treated with PatA, DMDA or vehicle for 48 h. Viability was determined by a formazan-based assay. The viability values of treated samples were related to vehicle. The experiment was performed with blood from three different donors. Two-way ANOVA with Dunnett's multiple comparisons was used to determine significant differences. * $p < 0.05$, ** $p < 0.01$, *** $p < 0.001$ indicate significant differences between elF4A inhibitor treated and vehicle treated samples. Abb. DMDA, des-methyl des-amino PatA; PatA, pateamine A.

Reference

1. Blum, L.; Gul, S.; Ulshofer, T.; Henke, M.; Krieg, R.; Berneburg, I.; Thomas, D.; Trautmann, S.; Kurz, J.; Geyer, J.; et al. In-vitro safety and off-target profile of the anti-parasitic arylmethylaminosteroid 1o. *Sci. Rep.* **2020**, *10*, 7534. https://doi.org/10.1038/s41598-020-64382-w.
